# Establishment of a Molecular Tumor Board (MTB) and Uptake of Recommendations in a Community Setting

**DOI:** 10.3390/jpm10040252

**Published:** 2020-11-27

**Authors:** Ari VanderWalde, Axel Grothey, Daniel Vaena, Gregory Vidal, Adam ElNaggar, Gabriella Bufalino, Lee Schwartzberg

**Affiliations:** West Cancer Center and Research Institute, Memphis, TN 38138, USA; agrothey@WESTCLINIC.com (A.G.); DVaena@WESTCLINIC.com (D.V.); gvidal@WESTCLINIC.com (G.V.); AElNaggar@WESTCLINIC.com (A.E.); GBufalino@WESTCLINIC.com (G.B.); lschwartzberg@WESTCLINIC.com (L.S.)

**Keywords:** MTB, precision medicine, cancer, oncology practice, community setting, CGP

## Abstract

In the precision medicine era, molecular testing in advanced cancer is foundational to patient management. Molecular tumor boards (MTBs) can be effective in processing comprehensive genomic profiling (CGP) results and providing expert recommendations. We assessed an MTB and its role in a community setting. This retrospective analysis included patients with MTB recommendations at a community-based oncology practice January 2015 to December 2018; exclusions were death within 60 days of the MTB and/or no metastatic disease. Potentially actionable genomic alterations from CGP (immunohistochemistry, in-situ hybridization, next-generation sequencing) were reviewed bi-weekly by MTB practice experts, pathologists, genetic counselors, and other support staff, and clinical care recommendations were provided. Subsequent chart reviews determined implementation rates of recommendations. In 613 patients, the most common cancers were lung (23%), breast (19%), and colorectal (17%); others included ovarian, endometrial, bladder, and melanoma. Patients received 837 actionable recommendations: standard therapy (37%), clinical trial (31%), germline testing and genetic counseling (17%), off-label therapy (10%), subspecialty multidisciplinary tumor board review (2%), and advice for classifying tumor of unknown origin (2%). Of these recommendations, 36% to 78% were followed by the treating physician. For clinical trial recommendations (*n* = 262), 13% of patients enrolled in a clinical trial. The median time between CPG result availability and MTB presentation was 12 days. A community oncology-based comprehensive and high-throughput MTB provided useful clinical guidance in various treatment domains within an acceptable timeframe for patients with cancer in a large community setting.

## 1. Introduction

Tumor biology is commonly driven by genomic alterations of oncogenic pathways that regulate processes such as cell differentiation, proliferation, apoptosis, and tumor metabolism [1]. Over the last 20 years, the development of targeted treatments against ‘actionable’ genomic changes has led to a paradigm shift towards precision medicine across several tumor types [1,2]. Molecular testing therefore allows for tailoring of treatment decisions based on the genomic makeup of a patient’s tumor. 

Expert consensus clinical practice guidelines have defined requirements for routine molecular testing in many malignancies, including advanced non-small cell lung cancer (NSCLC) [3,4], breast [5], ovarian [6,7], and colorectal cancers [8]. Given the number of different pathogenic mutations, comprehensive genomic profiling (CGP) can identify genomic drivers that are predictive of therapeutic response, and facilitate the timely implementation of treatment guidelines [9,10]. Furthermore, in some cancers, such as NSCLC, guidelines recommend use of CGP over multiple single-gene tests, and the FDA has approved the use of next-generation sequencing (NGS) testing panels [4,11]. However, cancer centers still have a number of clinical and operational considerations including choice of assay, laboratory, and analysis. Furthermore, the extensive tumor-specific molecular data produced from CGP can lead to uncertain clinical interpretation and utility. 

The sheer volume of diverse and continuously evolving data generated by CGP mean effective interpretation and application can be challenging for single-disease focused academic physicians or community oncologists (who are often generalists) [12]. However, molecular tumor boards (MTBs) with multidisciplinary input can provide an effective workflow and expert review process in order to generate precision medicine recommendations for oncology patients. However, implementation of an MTB within the community setting raises additional challenges: limited availability of genetics and genomics expertise, a lack of institutional imperative for an MTB, logistical barriers such as limited staff or meeting timings to ensure all specialties are attending, lack of dedicated expert physicians to screen cases and manage the MTB, and the need for timely submission of cases [13].

This report reviews the clinical utility of CGP and the role of an MTB in a large community oncology clinic on patient treatment decisions and outcomes. We also report the structure, organization, and management of the MTB, including best practices and lessons learned, analysis of patient management decisions based on CGP, and adherence to the MTB decisions.

## 2. Methods

### 2.1. Patients and Setting

A systematic program of CGP was initiated in December 2014 at the West Cancer Center, Memphis, TN, USA, a large community-based oncology practice. West Cancer Center physicians were encouraged to offer genomic testing to all patients with newly diagnosed metastatic melanoma, lung, colorectal, breast, and pancreatic cancer to a preferred third-party national testing laboratory. Other types of metastatic or recurrent malignancies were tested at the discretion of the treating provider.

### 2.2. CGP

Genomic profiling was largely conducted using the Caris MI^®^ Profile test (Caris Life Sciences, Phoenix, AZ, USA), which included multi-platform testing with chromogenic in-situ hybridization (CISH), NGS, and immunohistochemistry (IHC), provided commercially by Caris Life Sciences as part of standard of care testing. The NGS panel included 42 genes through 2015, after which a 592-gene panel was adopted for all samples (Supplementary Table S1). Additional relevant IHC testing was conducted on a tumor-lineage specific basis and could include PD-L1, estrogen receptor, progesterone receptor, androgen receptor, HER2, and others. Fusions were routinely detected using RNA-sequencing for ALK and ROS1, and CISH was used in breast and gastric cancer samples to measure HER2 overexpression.

Mutations were designated as pathogenic, presumed pathogenic, presumed benign, benign, or variant of unknown significance based on the interpretation of available genomic databases. Only pathogenic and presumed pathogenic mutations were reported unless otherwise noted. The criteria used for clinically actionable mutations are described in the Supplementary Material.

### 2.3. MTB

**Figure 1:**
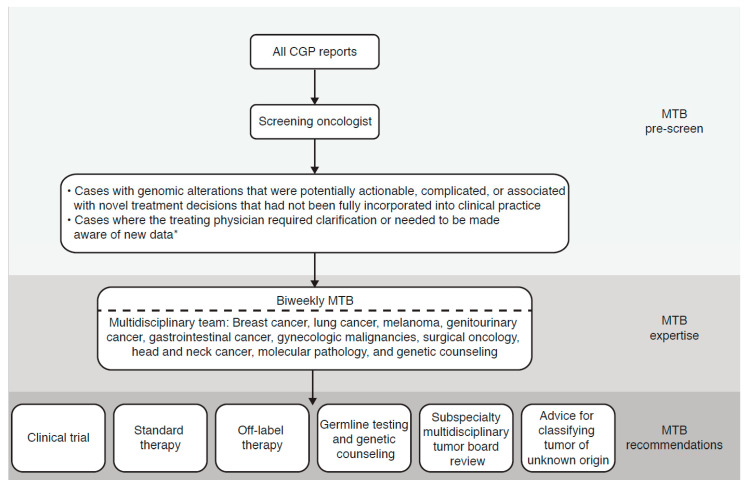
Molecular tumor boards (MTB) workflow and review process. * Data relevant to approvals and/or recommendations for the targeted therapy to be administered. CGP, comprehensive genomic profiling; MTB, molecular tumor board.

A bi-weekly, one-hour MTB meeting to review patient cases was set up (Figure 1). The MTB was designed to be comprehensive and high-throughput, with review of at least 50 cases bi-weekly prior to the meeting, and then full MTB review of 10 to 20 cases. All CGP reports were pre-screened for relevant genomic alterations by a MTB member through a testing laboratory-supported, online portal. While the policy was to obtain testing only for patients with newly diagnosed metastatic disease, reports were screened regardless of the timing of testing. Likewise, data from other genomic laboratories testing tissue or liquid biopsy samples were also screened if made available to the screening provider. Cases with genomic alterations that were potentially ‘actionable’, complicated, or associated with novel treatment decisions not fully incorporated into clinical practice were brought to the MTB for review. Cases could also be referred to the MTB if the treating physician specifically requested clarification of the case or if it was relevant to highlight new data regarding approvals and/or recommendations to the treating physician. Any other cases were omitted from the MTB.

MTB meetings were multi-specialty, multi-disciplinary, and open to any interested member of the center. Routine attendance consisted of medical and surgical oncologists with expertise in melanoma, breast, lung, head and neck, gastrointestinal, and genitourinary cancers, gynecologic malignancies, and surgical oncology. Other important members included anatomic and molecular pathologists and genetic counselors. Both the molecular data report and the clinical record from the electronic medical record (EMR) were reviewed and correlated at this live meeting, and the MTB provided recommendations for clinical care within the following six categories: (1) clinical trial; (2) standard therapy; (3) off-label therapy; (4) germline testing and genetic counseling; (5) subspecialty multidisciplinary tumor board review; and (6) advice for classifying tumor of unknown origin.

Recommendations were generally by consensus; any substantial disagreement among the MTB members was reflected in the recommendation summary. Minutes from the MTB were transcribed, input into the patient’s EMR, and an email with a summary and recommendations sent to the treating physician.

### 2.4. Analysis

MTB recommendations by category (Figure 1) and number of recommendations followed by the treating physician were collated and analyzed for all cases presented at the MTB from January 2015 through December 2018. Patients were excluded from the analysis if molecular testing did not meet clinical criteria or if the patient died within 60 days of the MTB, did not have metastatic disease, or was not given recommendations by the MTB.

## 3. Results

### 3.1. Patients and MTBs

Over the observation period, the MTB convened 92 times, of which 22, 21, 25, and 24 MTBs occurred in 2015, 2016, 2017, and 2018, respectively. As data were unavailable for four MTBs in 2015 and one MTB in 2018, the data reported include recommendations and follow-up from 87 MTBs. Of an estimated 4438 reported molecular test results, reports and clinical information from 837 patients were selected as potentially benefiting from additional clinical review at the MTB. Of these, 131 patients did not have a follow-up appointment and/or died within 60 days of the MTB, 54 patients did not have metastatic disease, 35 patients did not receive formal recommendations at the MTB, and 4 patients could not be found in the EMR database. As such, 613/4438 patients with recommendations were evaluated (14% of test reports) and received a total of 837 actionable recommendations. 

Of the 613 patients analyzed, 58% (*n* = 355) were female, African Americans comprised 30% (*n* = 181), with 68% *(n* = 417) Caucasian, and 2% (*n* = 15) other ethnicities. The most common cancers were lung (23%), breast (19%), and colorectal (17%). Other malignancies representing more than 20 cases each included ovarian, endometrial, and bladder cancers, and melanoma.

The median time between CGP results becoming available and presentation at the MTB was 12 days (interquartile range, 6 to 18 days). Median time from MTB presentation to last follow-up or date of death was 13.3 months. Among the 508 patients for whom extensive retrospective records were available, the median time from test ordering to result reporting (including time from order to biopsy, biopsy to pathology, pathology shipped to central test, processing, and resulting of the test) was 20 days.

### 3.2. MTB Recommendations and Adherence to Recommendations

**Figure 2:**
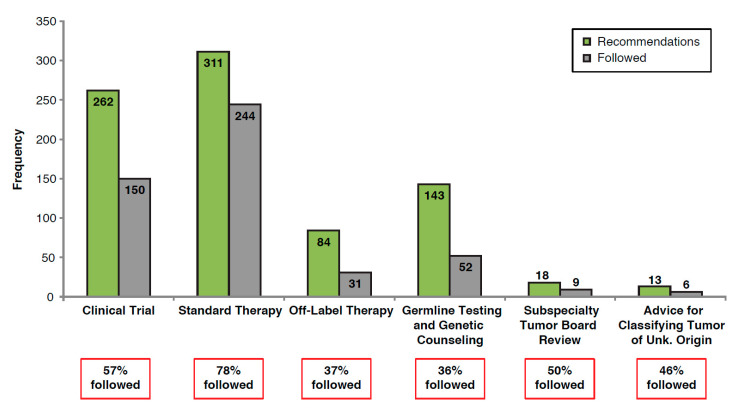
Proportion of MTB recommendations followed by the treating physician for all patients. MTB, molecular tumor board.

The majority of recommendations from the MTB were for standard therapy (51% of patients), and clinical trials (43% of patients; Table 1). Overall, recommendations followed by the treating physician ranged from 36% to 78%, depending on the category, with the highest compliance for following standard therapy (Figure 2).

MTB recommendations for clinical trial enrollment (*n* = 262) were followed by the treating physician for 150 (57%) patients, with 35 (13%) patients ultimately enrolling in a clinical trial. Of these clinical trial recommendations, the MTB recommended 167 (64%) patients to enroll immediately, of which 93 (56%) recommendations were followed by the treating physician and 20 (12%) patients enrolled in a trial (Supplementary Figure S1). The remaining MTB recommendations for clinical trial enrollment (*n* = 95; 36%) were for patients to enroll in future clinical trials upon progression, after standard of care therapy. Of these, 57 (60%) recommendations were followed and 15 (16%) patients enrolled in a clinical trial (Supplementary Figure S1). Among the 115 patients who did not follow the recommendation, the most common reasons for not enrolling included failed screening or ineligibility (34%), declined participation (18%), and lack of follow-up after a preliminary discussion (20%).

MTB recommendations for standard therapy and off-label therapy were followed by the treating physician in 78% (*n* = 244) and 37% (*n* = 31) of cases, respectively. Germline testing and genetic counseling recommendations were followed in 36% (*n* = 52) of cases; of these, 9 (17%) patients declined testing, did not follow-up, or were still deciding, 28 (54%) patients were negative, and 15 (29%) patients were positive for a germline mutation (ATM, *n* = 1; BRCA1, *n* = 2; BRCA2, *n* = 6; CHEK2, *n* = 2; Lynch syndrome, *n* = 2; homozygous MUTYH, *n* = 2). 

The type of recommendation given did not vary by race, with 31% of African American patients and 32% of Caucasian patients receiving the clinical trial recommendation; 40% and 36% were recommended standard therapy, 12% and 10% were recommended off-label therapy, and 15% and 18% were recommended germline testing, respectively. However, there appeared to be minor differences in the following of the recommendations, particularly in germline testing. Recommended germline testing was performed in 27% of African American patients compared with 38% of Caucasian patients. Similar numbers of African American and Caucasian patients followed recommendations for standard therapy (75% vs. 80%), off-label recommendations (32% vs. 39%) and clinical trials (52% vs. 60%); however, these did indicate slightly higher utilization of recommendations by Caucasian patients. 

### 3.3. The Value of MTBs beyond Standard Reporting

The following two cases illustrate how the MTB added value to therapeutic decisions, using combined knowledge of the tumor biology and clinical aspects of cases to guide treatment decisions; treatment suggestions based on molecular alterations would not be adequate in these cases.

In the first case, a 67-year-old woman with a BRCA2 germline mutation (exon 10 I605fs) had surgery for serous carcinoma of the fallopian tube and received adjuvant chemotherapy. She developed liver metastases and subsequently progressed on multiple therapies including endocrine therapy, poly ADP-ribose polymerase (PARP) inhibitors, immunotherapy, and chemotherapy. Following this, molecular analysis revealed new mutations. While circulating free DNA (cfDNA) testing revealed the known germline BRCA2 mutation, it also revealed BRCA2 G602fs (0.2%), BRCA2 c.794-16_797del (0.5%), and BRCA2 c.794-10_794-1del (0.8%). The latter two were described as splice-site indels and felt to represent ‘reversal’ mutations. Tumor molecular testing revealed the known exon 10 germline mutation but also an additional exon 10 mutation at p.G602E (16% variant frequency), which was described as a variant of uncertain significance and had not been previously present two years prior. Based on the combined data, the patient was referred to a clinical trial with a novel cell cycle regulating agent, to which she began responding. 

In the second case, a 49-year-old woman never-smoker was diagnosed with epidermal growth factor receptor (EGFR)-mutated (exon 19 p.E746_A750del) metastatic lung cancer four years prior to MTB presentation. After two years of treatment with EGFR-tyrosine kinase inhibitor (TKI), her tumor developed an EGFR T790M (exon 20) mutation, and she initiated osimertinib treatment. She responded for two years, but ultimately progressed. Repeat tissue molecular profiling showed the presence of a tertiary EGFR mutation (C797S, exon 20) which was notably not seen on liquid biopsy at the time and is associated with acquired resistance to osimertinib. MTB review indicated that similar combinations of mutations also conferred resistance to putative exon 20 inhibitors, but that combining osimertinib with a first-generation EGFR-TKI could possibly be an effective treatment option. However, the patient’s health declined as a result of disease progression, and she was unable to receive the recommended combination therapy.

## 4. Discussion

West Cancer Center was an early adopter of recommending routine CGP for advanced cancers and integrating an MTB to review the results in a timely fashion in a large clinical practice. Here we present four years of data in a pre-planned MTB in a large community setting, reflecting both a large sample size and a broad time-frame (>4400 reviewed reports and >800 patients reviewed in full MTBs). These results show that routine CGP with universal pre-screen review, selection of cases, and presentation at our high-throughput, bi-weekly MTB is feasible as a means of providing clinical guidance to a substantial proportion of cancer patients. 

Between 2015 and 2018, MTB recommendations followed by the treating physician ranged from 36% to 78% depending on the recommendation category, with the lowest rate of adherence to recommendations for genetic counseling and germline testing, and highest for standard therapy, similar to acceptance rates reported by other MTBs (27% to 70%) [14,15]. The low rate of germline testing is consistent with that seen in other centers and was likely multifactorial in etiology (patient refusal, provider non-prioritization, poor communication, etc.), although reasons for non-acceptance were not measured in our cohort [16].

The MTB was implemented to provide recommendations on clinical care, increase clinical trial participation, and improve awareness of standard or potential targeted therapies. We found MTB recommendations to be particularly useful in identifying clinical trial eligibility (recommended in 262 cases; 35 patients (13%) ultimately enrolled). This was slightly higher than the 7% rate reported in a community setting in Michigan [17], and similar to that reported by the Institut Curie Molecular Tumor Board, an academic medical center in a more centralized health care system where, among the 442 patients who underwent CGP, 10% were ultimately enrolled in a clinical trial [18]. Clinical trial enrollment in the present study was restricted by failed clinical trial screening, lack of follow-up after initial discussion regarding clinical trial enrollment, and patients declining participation. 

Our experience showed that turnaround times for molecular analysis and the delivery of recommendations were favorable in this community-based MTB compared with that reported in academic models. The median time from molecular diagnostic results being received and MTB patient presentation was 12 days. Moreover, overall turnaround time from time of request of molecular tests through to the provision of MTB recommendations was comparable with previous studies (median, 33 days) [19,20]. 

Rather than performing a ‘gate-keeper’ role [20], West Cancer Center MTB reviewed each case for appropriateness and the availability of tumor tissue prior to testing to avoid unnecessary testing and associated costs. In our institution, a clinic-wide policy dictated which cases should be sent for tissue-based CGP, namely common malignancies at first diagnosis of metastatic disease. Due to the timing of this approach, recommendations could be made for virtually all lines of therapy with the highest chance of identifying clinical trial opportunities while minimizing disruption to patients, as biopsies are performed at this time point for other clinical purposes. However, this approach can be problematic as molecular results may need to be recalled by the treating physician if the patient qualifies for a clinical trial as they progress through therapy. An additional challenge is that ‘upfront’ testing underestimates the future development of heterogeneous disease or acquired resistance mutations.

West Cancer Center MTB was innovative by adding a pre-screening step following the CGP report, allowing the MTB to focus only on those cases for which recommendations were of ultimate benefit to the patient. This is in contrast to other MTBs where physicians choose when and whether to present the case. Our methodology, therefore, created a high-throughput model where cases were not missed and maximum utility was maintained. Additionally, it led to cases being presented early in the clinical course, so that recommendations could take into account standard therapies for each disease and targeted actions after standard therapies failed.

There are several noteworthy limitations to our study. The number of ‘actionable’ molecular alterations reported may be conservative due to changes in actionability and the development of new targeted therapies over the period of the study (2015 to 2018), in addition to the evolution in genomic sequencing. Pre-screening of cases prior to presentation in the MTB was completed by a single physician and relied on their assessment of current clinic practices and baseline knowledge of individual physician practices, therefore the choice of which cases were ‘actionable’ was somewhat subjective and changed over time with the integration of new molecular-guided therapies into practice. This could have led to changes in what the screening physician decided could be considered of benefit to the treating physicians. We also acknowledge the financial implications involved in broad testing of patients; however, our study was designed to assess the role and feasibility of implementing an MTB in a community setting, and cost was not a key focus. 

As personalized/precision medicine becomes integrated into standard clinical practice, MTBs are playing an increasingly important role in supporting decision making by the treating physician. Robust, reproducible, and comprehensive bioinformatics analyses and close interactions between physicians and bioinformaticians are critical to the success of targeted therapy [21,22]. Our MTB was designed to be multi-omic, multi-specialty, and multi-disciplinary; we found that implementation of this comprehensive, collaborative approach led to increased clinical trial participation and more focused use of off-label targeted therapy within our institution. Moreover, the MTB meetings provided an educational opportunity and facilitated increased awareness among physicians of targeted therapies to match genomic alterations. 

In conclusion, our comprehensive and high-throughput bi-weekly MTB was feasible as a means to providing clinical guidance within an acceptable timeframe for patients with cancer in a large community setting. It may therefore be useful to adopt similar models in other cancer centers.

## Figures and Tables

**Table 1:** Recommendations by category from the MTB for all patients analyzed (*n* = 613).

Recommendation Category	Number of Recommendations	Proportion of Patients Receiving Recommendations ^1^ (%)
Clinical trial	262	43
Standard therapy	311	51
Off-label therapy	84	14
Germline testing and genetic counseling	143	23
Subspecialty multidisciplinary tumor board review	18	3
Advice for classifying tumor of unknown origin	13	2

^1^ Patients could have more than one recommendation. Abbreviations: MTB, molecular tumor board.

## Supplementary Materials

The following are available online at https://www.mdpi.com/2075-4426/10/4/252/s1, Figure S1: MTB recommendations for clinical trial enrollment and resulting patient management, Table S1: Caris MI^®^ gene panel.
