# Supplementary material for: Establishment of a Molecular Tumor Board (MTB) and Uptake of Recommendations in a Community Setting
Source: J Pers Med. 2020 Nov 27;10(4):252. doi: 10.3390/jpm10040252 (PMC7711773; doi:10.3390/jpm10040252)
Supplement: Supplementary file 1 [file jpm-10-00252-s001.pdf]

SUPPLEMENTARY MATERIAL

The criteria for clinically-actionable mutations in order of decreasing clinical validity were:

- Approved drug for the alteration with a well-defined biomarker in that disease
- Approved drug for the alteration, which was well defined in another disease (ie, vemurafenib approved for BRAF V600E mutation in melanoma, applied to non-small cell lung cancer
- Evidence for a benefit of a drug with well-defined biomarkers from reported clinical trials in a specific disease
- A drug was being studied in a clinical trial in one disease or multiple diseases linked to a biomarker
- A drug was available with a case report of response (n = 1)
- A drug was available that had demonstrated pre-clinical activity against that alteration in a specific disease.

Supplementary Table 1. Caris MI® gene panel

Next-Generation Sequencing – Genomic Stability Testing (DNA)

Microsatellite Instability (MSI)	Tumor Mutational Burden (TMB)*

Next-Generation Sequencing – Point Mutations and Indels (DNA)

ABI1	BRD4	CRLF2	FOXO4	HOXC11	KLF4	MUC1	PAK3	RHOH	TAL2
ABL1	BTG1	DDB2	FSTL3	HOXC13	KLK2	MUTYH	PATZ1	RNF213	TBL1XR1
ACKR3	BTK	DDIT3	GATA1	HOXD11	LASP1	MYCL (MYCL1)	PAX8	RPL10	TCEA1
AKT1	C15orf65	DNM2	GATA2	HOXD13	LMO1	NBN	PDE4DIP	SEPT5	TCL1A
AMER1 (FAM123B)	CBLC	DNMT3A	GNA11	HRAS	LMO2	NDRG1	PHF6	SEPT6	TERT
AR	CD79B	EIF4A2	GPC3	IKBKE	MAFB	NKX2-1	PHOX2B	SFPQ	TFE3
ARAF	CDH1	ELF4	HEY1	INHBA	MAX	NONO	PIK3CG	SLC45A3	TFPT
ATP2B3	CDK12	ELN	HIST1H3B	IRS2	MECOM	NOTCH1	PLAG1	SMARCA4	THRAP3
ATRX	CDKN2B	ERCC1	HIST1H4I	JUN	MED12	NRAS	PMS1	SOCS1	TLX3
BCL11B	CDKN2C	ETV4	HLF	KAT6A (MYST3)	MKL1	NUMA1	POU5F1	SOX2	TMPRSS2
BCL2	CEBPA	FAM46C	HMGN2P46	KAT6B	MLLT11	NUTM2B	PPP2R1A	SPOP	UBR5
BCL2L2	CHCHD7	FANCF	HNF1A	KCNJ5	MN1	OLIG2	PRF1	SRC	VHL
BCOR	CNOT3	FEV	HOXA11	KDM5C	MPL	OMD	PRKDC	SSX1	WAS
BCORL1	COL1A1	FOXL2	HOXA13	KDM6A	MSN	P2RY8	RAD21	STAG2	ZBTB16
BRD3	COX6C	FOXO3	HOXA9	KDSR	MTCP1	PAFAH1B2	RECQL4	TAL1	ZRSR2

Next-Generation Sequencing – Point Mutations, Indels and Copy Number Alterations* (DNA)

ABL2	BRCA1	CREB3L1	ETV1	GAS7	KMT2A (MLL)	MYCN	PER1	RUNX1	TFEB
ACSL3	BRCA2	CREB3L2	ETV5	GATA3	KMT2C (MLL3)	MYD88	PICALM	RUNX1T1	TFG
ACSL6	BRIP1	CREBBP	ETV6	GID4 (C17orf39)	KMT2D (MLL2)	MYH11	PIK3CA	SBDS	TFRC
ADGRA2	BUB1B	CRKL	EWSR1	GMPS	KNL1	MYH9	PIK3R1	SDC4	TGFBR2
AFDN	CACNA1D	CRTC1	EXT1	GNA13	KRAS	NACA	PIK3R2	SDHAF2	TLX1
AFF1	CALR	CRTC3	EXT2	GNAQ	KTN1	NCKIPSD	PIM1	SDHB	TNFAIP3
AFF3	CAMTA1	CSF1R	EZH2	GNAS	LCK	NCOA1	PML	SDHC	TNFRSF14
AFF4	CANT1	CSF3R	EZR	GOLGA5	LCP1	NCOA2	PMS2	SDHD	TNFRSF17
AKAP9	CARD11	CTCF	FANCA	GOPC	LGR5	NCOA4	POLE	SEPT9	TOP1
AKT2	CARS	CTLA4	FANCC	GPHN	LHFPL6	NF1	POT1	SET	TP53
AKT3	CASP8	CTNNA1	FANCD2	GRIN2A	LIFR	NF2	POU2AF1	SETBP1	TPM3
ALDH2	CBFA2T3	CTNNB1	FANCE	GSK3B	LPP	NFE2L2	PPARG	SETD2	TPM4
ALK	CBFB	CYLD	FANCG	H3F3A	LRIG3	NFIB	PRCC	SF3B1	TPR
APC	CBL	CYP2D6	FANCL	H3F3B	LRP1B	NFKB2	PRDM1	SH2B3	TRAF7
ARFRP1	CBLB	DAXX	FAS	HERPUD1	LYL1	NFKBIA	PRDM16	SH3GL1	TRIM26
ARHGAP26	CCDC6	DDR2	FBXO11	HGF	MAF	NIN	PRKAR1A	SLC34A2	TRIM27
ARHGEF12	CCNB1IP1	DDX10	FBXW7	HIP1	MALT1	NOTCH2	PRRX1	SMAD2	TRIM33
ARID1A	CCND1	DDX5	FCRL4	HMGA1	MAML2	NPM1	PSIP1	SMAD4	TRIP11
ARID2	CCND2	DDX6	FGF10	HMGA2	MAP2K1 (MEK1)	NR4A3	PTCH1	SMARCB1	TRRAP
ARNT	CCND3	DEK	FGF14	HNRNPA2B1	MAP2K2 (MEK2)	NSD1	PTEN	SMARCE1	TSC1
ASPSCR1	CCNE1	DICER1	FGF19	HOOK3	MAP2K4	NSD2	PTPN11	SMO	TSC2
ASXL1	CD274 (PDL1)	DOT1L	FGF23	HSP90AA1	MAP3K1	NSD3	PTPRC	SNX29	TSHR
ATF1	CD74	EBF1	FGF3	HSP90AB1	MCL1	NT5C2	RABEP1	SOX10	TTL
ATIC	CD79A	ECT2L	FGF4	IDH1	MDM2	NTRK1	RAC1	SPECC1	U2AF1
ATM	CDC73	EGFR	FGF6	IDH2	MDM4	NTRK2	RAD50	SPEN	USP6
ATP1A1	CDH11	ELK4	FGFR1	IGF1R	MDS2	NTRK3	RAD51	SRGAP3	VEGFA
ATR	CDK4	ELL	FGFR1OP	IKZF1	MEF2B	NUP214	RAD51B	SRSF2	VEGFB
AURKA	CDK6	EML4	FGFR2	IL2	MEN1	NUP93	RAF1	SRSF3	VTI1A
AURKB	CDK8	EMSY	FGFR3	IL21R	MET	NUP98	RALGDS	SS18	WDCP
AXIN1	CDKN1B	EP300	FGFR4	IL6ST	MITF	NUTM1	RANBP17	SS18L1	WIF1
AXL	CDKN2A	EPHA3	FH	IL7R	MLF1	PALB2	RAP1GDS1	STAT3	WISP3
BAP1	CDX2	EPHA5	FHIT	IRF4	MLH1	PAX3	RARA	STAT4	WRN
BARD1	CHEK1	EPHB1	FIP1L1	ITK	MLLT1	PAX5	RB1	STAT5B	WT1
BCL10	CHEK2	EPS15	FLCN	JAK1	MLLT10	PAX7	RBM15	STIL	WWTR1
BCL11A	CHIC2	ERBB2 (HER2/NEU)	FLI1	JAK2	MLLT3	PBRM1	REL	STK11	XPA
BCL2L11	CHN1	ERBB3 (HER3)	FLT1	JAK3	MLLT6	PBX1	RET	SUFU	XPC
BCL3	CIC	ERBB4 (HER4)	FLT3	JAZF1	MNX1	PCM1	RICTOR	SUZ12	XPO1
BCL6	CIITA	ERC1	FLT4	KDM5A	MRE11	PCSK7	RMI2	SYK	YWHAE
BCL7A	CLP1	ERCC2	FNBP1	KDR (VEGFR2)	MSH2	PDCD1 (PD1)	RNF43	TAF15	ZMYM2
BCL9	CLTC	ERCC3	FOXA1	KEAP1	MSH6	PDCD1LG2 (PDL2)	ROS1	TCF12	ZNF217
BCR	CLTCL1	ERCC4	FOXO1	KIAA1549	MSI2	PDGFB	RPL22	TCF3	ZNF331
BIRC3	CNBP	ERCC5	FOXP1	KIF5B	MTOR	PDGFRA	RPL5	TCF7L2	ZNF384
BLM	CNTRL	ERG	FUBP1	KIT	MYB	PDGFRB	RPN1	TET1	ZNF521
BMPR1A	COPB1	ESR1	FUS	KLHL6	MYC	PDK1	RPTOR	TET2	ZNF703
BRAF	CREB1								

Whole Transcriptome Sequencing – Genes most commonly associated with cancer listed below.

Fusions (RNA)								Variant Transcripts (RNA)
ABL	BRD3	FGFR3	INSR	MYB	NUMBL	PRKCA	RSPO3	AR-V7
AKT3	BRD4	ERG	MAML2	NOTCH1	NUTM1	PRKCB	TERT	
ALK	EGFR	ESR1	MAST1	NOTCH2	PDGFRA	RAF1	TFE3	EGFR vIII
ARHGAP26	EWSR1	ETV1	MAST2	NRG1	PDGFRB	RELA	TFEB	
AXL	FGR	ETV4	MET	NTRK1	PIK3CA	RET	THADA	
BCR	FGFR1	ETV5	MSMB	NTRK2	PKN1	ROS1	TMPRSS2	MET Exon 14 Skipping
BRAF	FGFR2	ETV6	MUSK	NTRK3	PPARG	RSPO2		

Supplementary Fig 1. MTB recommendations for clinical trial enrollment and resulting patient management.
